# Soft Tissue Contour Impression with Analogic or Digital Work Flow: A Case Report

**DOI:** 10.3390/ijerph15122623

**Published:** 2018-11-23

**Authors:** Luigi Canullo, Andrea Di Domenico, Fabio Marinotti, Maria Menini, Paolo Pesce

**Affiliations:** 1 Private Practice, Via Nizza, 46, 00198 Rome, Italy; marinottifabio@yahoo.it; 2 Private Practice, Via Benincasa, 11, 84013 Cava de’ Tirreni, Italy; adido8@hotmail.com; 3 Department of Surgical Sciences (DISC), Implant and Prosthetic Dentistry Unit, University of Genoa, Ospedale S. Martino (Pad. 4), L. Rosanna Benzi 10, 16132 Genoa, Italy; maria.menini@unige.it (M.M.); paolo.pesce@unige.it (P.P.)

**Keywords:** digital dentistry, esthetics, digital work-flow, analogic workflow, bone augmentation, implant dentistry, PES, radiographic analysis

## Abstract

Purpose: Transferring precise information to the dental laboratory is one of the key factors to achieving clinical success. The aim of the present study was to describe classical and digital work-flows used to rehabilitate an implant with a convergent collar in the aesthetic zone following the BOPT (biologically oriented preparation technique) approach and to report the three years follow-up outcomes of two patients rehabilitated following such procedures. Materials and methods: Two central incisors of two different patients were rehabilitated with a tissue-level implant with a convergent collar and, after a provisional and healing phase, one implant was “impressed” using a classical workflow and one using a digital one. The primary outcome measured was the mean bone loss. An intraoral radiograph was taken at crown delivery and at the three years follow-up visit. Secondary Esthetic outcomes pre-op and post-op were evaluated using the PES (pink esthetic score). Results: At the three years follow-up visit, radiographic analysis showed no signs of bone resorption. For the analogic procedure, the pre-and post-op PES scores were 8, whereas for the digital procedure the pre-op PES score was 4 and a post-op score of 9 was obtained. Conclusions: classical and digital work flows succeeded in giving precise and complete anatomical information of implant position, including the soft tissue contour. Minimum bone loss and an esthetic success were obtained in both procedures.

## 1. Introduction

In recent years, the management of the esthetic areas and the maintenance of tissue stability surrounding implant abutment have been two of the main topics of discussion and study [1,2].

Bacterial infiltration at the implant–abutment connection has been claimed to be responsible of crestal bone loss, with possible consequential onset of an inflammatory process [3]. Different techniques and components morphologies have been tested to improve tissue stability and reduce bone loss and inflammation around the implant–abutment connection. The Platform-Switching concept (PS) has been clinically validated by the literature [4] The aim is moving the bacterial infiltration at the implant–abutment interface far from crestal bone towards the centre of the implant, thus reducing the marginal bone loss. 

On the other side, transmucosal implants have been proposed that move the implant–abutment connection coronally with respect to crestal bone. However, this design remains controversial [5]. In fact, the classical transmucosal implant neck design presents a divergent morphology making difficult the clinical management of the tissues especially in the esthetic areas [6].

Recently, a prosthetic protocol (BOPT—biologically oriented preparation technique) has been proposed by Ignazio Loi [7] for the vertical preparation of natural teeth, with the aim to module soft tissue compression through provisional crowns and maximize soft tissue upgrowth.

The BOPT principles have been transposed in implant dentistry, creating a transmucosal implant with a coronally convergent neck. This convergent neck allows the realization of cemented fixed restorations following the same BOPT principles applied when restoring natural teeth with a vertical preparation. In fact, the crown emergence forms a continuity between the implant and the prosthodontics components without any horizontal gap [8]. In this way, the infiltrated bacteria are sealed inside the prosthodontic components and maintained away from the bone.

This design makes it easier for the clinician soft tissues conditioning and makes peri-implant soft tissue management more similar to the management of periodontal tissues in tooth-supported restorations as opposed to traditional implant abutments or divergent neck tissue-level implants. Once peri-implant tissues are healed, one of the key factors in order to achieve an aesthetic success is the transfer to the dental lab of a precise impression of the “shaped” provisional crown and of peri-implant soft tissues. In particular, the impression of the soft tissue contour is particularly demanding.

Impressions-taking procedures are rapidly evolving and new technologies may aid the clinicians in data transfer to the dental laboratory [9]. In particular, traditional impression materials and techniques are more and more replaced by intraoral scanning (IOS) followed by CAD/CAM-production of anatomically full-contour restorations or frameworks combined with CAD-on veneering [10].

The aim of the present study was to describe classical and digital work-flows used to rehabilitate a tissue-level implant with a convergent collar in the aesthetic zone following the BOPT approach and to report the three years follow-up outcomes of two patients rehabilitated following such procedures.

## 2. Materials and Methods

The present report was conducted in accordance with the Helsinki Declaration. All patients were carefully informed about the study protocol and provided written informed consent prior to the start of the study.

### 2.1. Study Design and Patient Enrollment

Between January and June 2015, two patients from a private dental office in Rome were enrolled. Patients were in good medical conditions, with no contraindications to oral surgery and with one hopeless central incisor tooth in the maxillary area. One patient, rehabilitated with the analogical work-flow was 39 years old, male; the other, rehabilitated with a digital work flow, was 48 years old, male.

Both patients were non-smokers, did not assume anticoagulants, and were not immunosuppressed. Before implants insertion patients underwent scaling, root planning, and received oral hygiene instructions or any periodontal treatment necessary. Both patients were treated by the same clinician (L.C.). Hopeless teeth were extracted using a minimally invasive, flapless approach and Mg-enriched nano-hydroxyapatite (Mg-e HA) granules (SINTlife 600–900 μm, Finceramica) were grafted into the socket up to 3 mm apical to the soft tissue margin. A collagen disk (Gingistat, GABA-Vebas) was used to seal the alveolar socket and a Maryland bridge was adopted as provisional restoration.

After three months, a re-entry procedure was performed and two identical implants (length: TOT mm, diameter: TOT mm) were inserted after mini-flap elevation. The implants (Prama, Sweden & Martina, Padova, Italy) used had a machined titanium, 2.8 mm high, cylindrical/conical shape collar, tapered in occlusal direction. The platform presents an internal hexagon connection with a 3.4 mm diameter.

The implant body-collar interface was positioned 0.5 mm below the level of the bone crest and the implant was mid-crestally inserted. In fact, the cylindrical part of the smooth collar (0.8 mm) was positioned almost below the bone level. Attention was paid that at least 1.5 mm of bone volume was present all around the implant coronal platform.

Before surgery, impressions of the dental arches were obtained and a resin provisional crown was prepared to fabricate a chairside cemented provisional restoration.

**Figure 1:**
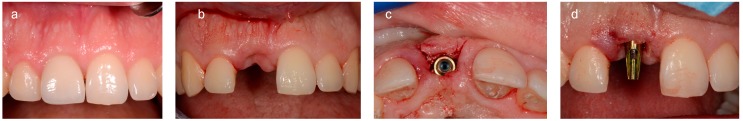
Preoperative situation of first case (**a**) and soft tissue contour after site development 3 months after tooth extraction (**b**); implant insertion (**c**); provisional abutment screwed immediately after implant insertion (**d**).

**Figure 2:**
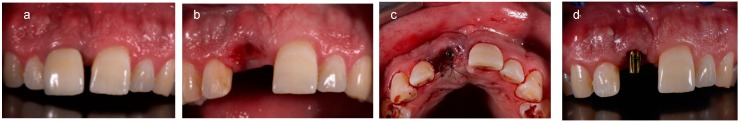
Preoperative situation of second case(**a**) (please note the interincisal diastema) and soft tissue contour after site development 3 months after tooth extraction (**b**); implant insertion (**c**); provisional abutment screwed immediately after implant insertion (**d**).

**Figure 3:**
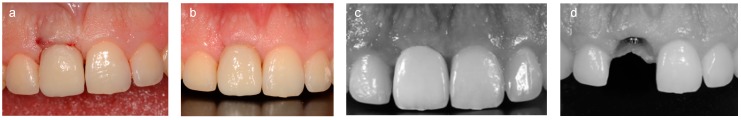
Soft tissue contour of first case after different provisional restoration modifications 4 and 1 months after implant insertion (**a**–**b**); soft tissue contour after soft tissue maturation with and without provisional restoration after one additional month(**c**–**d**).

**Figure 4:**
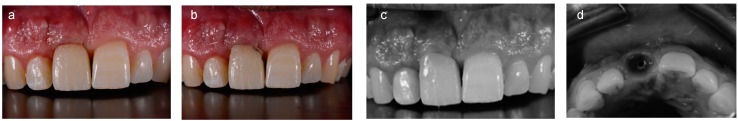
Soft tissue contour of the second case after different provisional restoration modifications 1 month after implant insertion (**a**–**b**); soft tissue contour after soft tissue maturation with and without provisional restoration additional 1 month after implant insertion (**c**–**d**).

A temporary titanium abutment was directly screwed on the implant and the provisional crown was adjusted avoiding occlusal contacts. The provisional crown was shaped and remodeled over time according to the principles of BOPT technique [10] and circumferentially embraced the smooth surfaced convergent collar of the implant. Taking advantage of the transgingival convergent neck of the implant, the margin line and the emergence profile were individually designed to improve the esthetics of soft tissues. The provisional crown was cemented using a provisional cement (Figure 1 and Figure 2). Three months thereafter, tissues were ready for the final impression (Figure 3 and Figure 4) and two different work-flows were followed in the two patients:

### 2.2. Analogic Work-Flow

**Figure 5:**
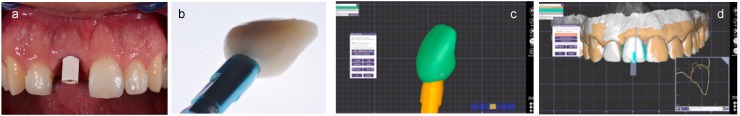
The first case: scan abutment at the time of digital impression (**a**) and the provisional crown with the abutment and analog (**b**); scanning of the final provisional restoration defining the sub-gingival contour (**c**); matching of the models and the provisional restoration (**d**).

An individualized transfer was created, that was modeled according to the provisional crown as described by Papadopoulos et al. [11]. A silicon open tray impression was taken in order to give to the dental lab all the information about soft peri-implant tissues and the final master cast was produced (Figure 5).

### 2.3. Digital Work-Flow

Three intraoral impressions were obtained using an intraoral scanner (CS3600, Carestream, Atlanta, GA, USA):

1. Dental arch with the cemented provisional crown,
2. Provisional crown connected with a digital analogue,
3. Dental arch with a scan body screwed onto the implant.

**Figure 6:**
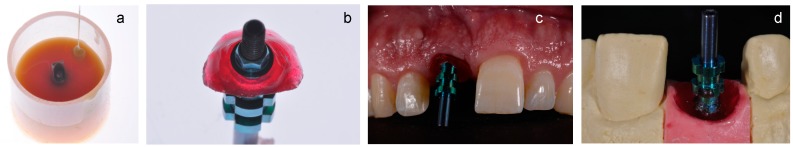
Second case Sub-gingival provisional restoration contour duplication (**a**); Modification of the transfer to reproduce subgingival contour (**b**); traditional analogic impression with modified pick-up transfer (**c**); transfer of the soft tissue contour on the model (**d**).

All these information were matched, transferring to the dental lab both the provisional crown shape and soft tissues contour. The “digital” master cast was produced (Figure 6).

Both patients were rehabilitated with a CAD/CAM zircon abutment and a final lithium disilicate CAD/CAM crown realized by the same dental technician and luted using a resin cement.

### 2.4. Outcome Measures

The primary outcome measured was mean bone loss. Patients underwent a standardized periapical radiograph at crown delivery and at the three-year follow-up visit. Radiographs were obtained with the parallel long-cone technique. The implant body-collar interface was used as a reference point for bone level measurements on mesial and distal side of each implant. Two examiners (P.P. and M.M.) performed the clinical measurements after a calibration exercise demonstrating 95.7% concordance within ±0.5 mm for measurements.

**Figure 7:**
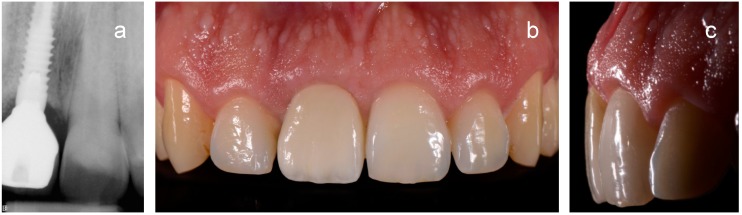
Definitive restoration of the first case after three years: radiographic (**a**) and clinical appearance (**b**,**c**).

**Figure 8:**
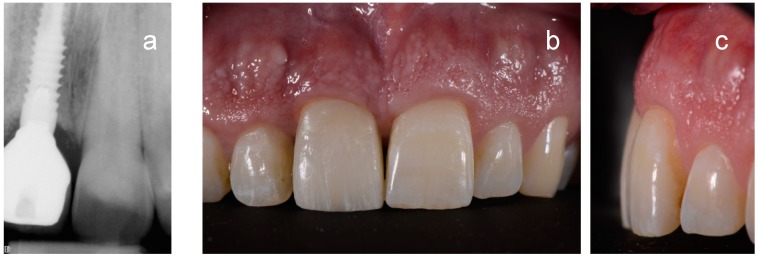
Definitive restoration of the second case after three years: radioraphic (**a**) and clinical appearance (**b**,**c**).

Secondary Esthetic outcomes were evaluated using PES (pink esthetic score) modified by Belser et al. and comparing pre- and post-operative situation for each scenario. A score of 2, 1, or 0 was assigned to all five PES parameters. The sum of the five adds up, under optimum conditions, to a score of 10. Photographs were captured with Canon Rebel XT equipped with 100-mm macro with ring flash, under similar light conditions. All measurements were made by two independent examiners using photographs available in Power Point files (P.P. and M.M.). (Figure 7 and Figure 8).

During the three-year follow-up, appointments for professional oral hygiene were scheduled every 6 months.

## 3. Results

Both patients attended scheduled follow-up appointments and at the three years follow-up visit both implants were in function and stable. Radiographic analysis showed no signs of bone resorption at three years. Mean bone loss for the implant rehabilitated with the analogic procedure was 0.08 mm, whereas for the digital procedure it was 0.05 mm.

Esthetic comparison of pre- vs. post-op situation at the three years of follow-up showed a pre-op PES score of 8 and a post-op score of 8 for the analogic procedure, whereas for the digital procedure pre-op PES score was 4 and a post-op score 9.

Both patients referred to be satisfied of the esthetic appearance of their implant rehabilitation.

## 4. Discussion

The clinical application of digital workflows in dentistry has increased in recent years due to the headway made in technologies such as intraoral scanners and software, which have improved communication and data transfer between the clinician and the dental technician [10].

Additionally, digital impression techniques consent to overcome some drawbacks of conventional impressions such as: discomfort, nausea, unsatisfactory taste, time consumption, remakes in case of air bubbles inclusion, and forceful removal of highly retentive impressions with a risk for potential damage [12].

Digital impression is becoming widespread in clinical dental practice and it is considered equally accurate as conventional impressions for individual restorations or 3–4 element bridges on natural teeth and on implants [13,14] or even implant full-arch restorations [15]. 

Conversely the main disadvantages of using digital impressions are: the difficulty in detecting deep margin lines in prepared teeth and in case of bleeding, the learning curve, and the purchasing and managing costs [13,16].

The present research described a conventional and a digital workflow used to rehabilitate a tissue-level implant with a convergent collar in the aesthetic zone. Following the BOPT technique, the position of the soft tissues and the final crown esthetic is determined by the crown’s contours [7,8], therefore the management of the provisional crown is of paramount importance in shaping tissues. At the same time, transferring all the information about the provisional crown and the shaped peri-implant soft tissues to the dental lab is the key to achieve aesthetic success.

Joda et al. [17] described the “Individualized Scanbody Technique” to transfer the mucosal information to the dental lab, individually modifying the scan body in accordance with the created emergence profile of the provisional implant-supported restoration. In this way, the mucosa outline can be transferred predictably into the process chain of the analogical workflow. With the digital work-flow described in the present paper this passage has been overcome, in fact the technician was directly provided with the scannerization of the provisional restoration. The technique presented in this case report might results slightly more precise compared to the one described by Joda. In fact, the one reported in the present study allows a better matching because it is based on the original provisional restoration, avoiding any intermediate step for the duplication of the mucosal profile.

Comparing the two workflows, both succeeded in giving the dental lab precise and complete information about the implant position, including the soft tissue contour, to realize a clinically successful final crown. 

It must be underlined that the present paper describes a case series of two patients only. Further comprehensive and long-term clinical research is needed to support the present observations.

## 5. Conclusions

Within the limits of the present case report, analogic and digital work flows succeeded in giving precise and complete anatomical information of implant position, including the soft tissue contour. Results after three years showed minimum bone loss and an esthetic success in both procedures.
